# A case report of severe adverse reaction of exenatide: Anaphylactic shock

**DOI:** 10.1097/MD.0000000000030805

**Published:** 2022-09-30

**Authors:** Xujing Liu, Aihua Zhai, Bai Zhang

**Affiliations:** a Department of Clinical Laboratory, the Fifth People’s Hospital of Jinan, China; b Department of Pharmacy, the Fifth People’s Hospital of Jinan, China.

**Keywords:** anaphylactic shock, anaphylaxis, exenatide

## Abstract

Anaphylactic shock is the severe state of the allergic reaction, which is rapid in onset and fatal. This is the first study that discusses the anaphylactic shock of exenatide reexposure in the patient who has interrupted exenatide treatment.

**Patient concerns:**

A 47-year-old man was treated with exenatide owing to high blood glucose and obesity. Then he developed localized urticarial on the face, white lip, hands tremble, nausea, vomit, chest stuffiness, dizziness, accompanying with confusion and dyspnea. His blood glucose was 4.6 millimole per liter (mmol/L) and blood pressure was 85/50 millimeters of mercury (mm Hg).

**Diagnosis:**

Exenatide-induced anaphylactic shock was considered.

**Interventions:**

The emergency electrocardiogram was performed. The patient was treated with dexamethasone sodium phosphate and calcium gluconate, combined with exenatide withdrawal. He also received oral antiallergic agents and intravenous nutrition treatment.

**Outcomes:**

After antishock treatment, the clinical response gradually alleviated.

**Lessons:**

Although exenatide is not prone to anaphylaxis, it is the synthetic peptide that can induce antibody formation. Exenatide has immunogenicity with the potential to elicit an allergic reaction upon administration. Clinicians should always pay more attention to the anaphylactic shock of exenatide, when prescribing for diabetics.

## 1. Introduction

Exenatide is a glucagon-like peptide-1 receptor agonist, which is the first novel incretin analogues with natural glucagon-like peptide-1 activity for treatment of type 2 diabetes.^[1]^ The common adverse effects of exenatide reported in patients include gastrointestinal disorders, pancreatic damage, hypoglycemia, and injection-site reactions. However, anaphylaxis induced by exenatide is very rare (<1/10,000).^[2]^ Anaphylactic shock is the severe form of anaphylaxis, and it is the fast developing and potentially fatal systemic reaction.

## 2. Case report

On March 25, 2016, a 47-year-old man was admitted to hospital owing to high blood glucose and urine protein revealed in physical examination. He had personal history of type 2 diabetes for 14 months and urine protein for one month treated with antidiabetic drugs irregularly. The patient also suffered from high blood pressure for over 4 years with the peak blood pressure at 170/120 millimeters of mercury (mm Hg). He was able to keep his blood pressure at about 140/100 mm Hg with irbesartan and aspirin orally. No history of drug or food allergies was found. The patient has provided informed consent for publication of the case.

The patient had a body mass index of 37.8 kg/m^2^ with height 181cm and weight 124 kg. He had been treated with exenatide since the diagnosis of diabetes. The initial dose was 5 μg subcutaneously twice daily (b.i.d.), and the subsequent maintenance dose was 10 μg subcutaneously b.i.d. The patient was discharged after stable control of blood glucose and blood pressure. He continued to take exenatide, but self-discontinued it due to scleroma at the injection site. Without any other treatment, the symptom was relieved, therefore, we failed to be informed. When the patient was admitted again because of diabetes, we administrate the hypoglycemic (exenatide 10 μg subcutaneously b.i.d, metformin hydrochloride sustained release tablets 500 mg orally b.i.d. and acarbose 50 mg orally 3 times daily), antihypertensive (irbesartan 150 mg orally once daily) and antiplatelet (aspirin enteric-coated tablets 100 mg orally once daily) agents. On March 29, 2016, exenatide was added at a dose of 10 μg in the morning and 20 μg in the evening due to poor blood glucose control, combining with the severe obesity. At 16:30 on April 5, exenatide was given, and half an hour later, he developed generalized urticarial. The symptom was gradually relieved after administration of loratadine tablets. At 6:40 in the morning of 7 of April, he developed localized urticarial on the face, white lip, hands tremble, nausea, vomit, chest stuffiness, dizziness, accompanying with confusion and dyspnea. His blood glucose was 4.6 mmol/L and blood pressure was 85/50 mm Hg. There were no abnormal findings by emergency electrocardiogram. Dexamethasone sodium phosphate and calcium gluconate were commenced. Upon treatment, his blood glucose was 8.6mmol/L and blood pressure was 120/102 mm Hg. However, He still felt nausea, vomiting, dizziness and discomfort. Because the above symptoms were induced after the injection of exenatide, he received treatment with oral hypoglycemic agents instead of exenatide, accompanying with oral antiallergic agents and intravenous nutrition treatment. The clinical response gradually alleviated and the patient’s mood was calmed down. All these clinical symptoms and examinations provide an assistance for confirming the diagnosis of exenatide-induced anaphylactic shock.

## 3. Discussion

In our study, the patient suffered urticarial, and sequently anaphylactic shock after exenatide injection. Anaphylactic shock is the severe state of the allergic reaction, which is rapid in onset, and if not handled promptly, sometimes it can prove fatal. Within half an hour after the injection of exenatide, the patient suffered anaphylactic shock which exhibits cardiovascular symptoms such as drop of blood pressure to 85/50 mm Hg, chest tightness, dizziness, and confusion. With respect to the gastrointestinal tract, the patient displayed typical symptoms like nausea and vomiting. The abnormal performance of the anaphylactic reaction was clearly correlated with the injection time of exenatide. The symptoms develop immediately (half an hour) after exposure to the specific allergen (exenatide). The yinxin damo injection and kudie zi injection had been administrated to the patient while hospitalized. However, no intravenous drug was administered prior to the onset of anaphylaxis induced by exenatide. Moreover, the allergic reactions of oral drugs including metformin hydrochloride sustained release tablets, acarbose tablets, irbesartan tablets, aspirin enteric-coated tablets, shiwei longdan hua particles, and qingyan dropping pill are very rare or not mentioned in the specification. And the administration time was not consistent with occurrence of allergy symptoms, which can basically exclude the interference of these drugs. There were no other abnormalities pre- and post- medication and no exposure to other allergens. Therefore, the patient was treated with an intermittent therapy of exenatide, which was helpful in confirming the diagnosis of exenatide-induced anaphylactic shock.

Although exenatide is not prone to anaphylaxis, it is the synthetic peptide originally isolated from the Heloderma suspectum lizard, which can induce antibody formation that is commonly present in therapeutic peptides.^[3]^ When the patients were treatment by exenatide, anti-exenatide antibodies were more common. Low antibody titers do not appear to impacting the efficacy of exenatide, however, high antibody titers in a small percentage of patients reduced mean efficacy, which was statistically significant in the group with exenatide treatment once a week. Anti-exenatide antibodies also associated with the injection site reactions.^[4]^ Numerous research studies reported that antibody formation may induce several consequences,^[5,6]^ it is supposed that exenatide has immunogenicity with the potential to elicit an allergic reaction upon administration. We have retrieved all the papers that are published in *the pubmed* up to now and found that there were only 3 reports referred for the systemic hypersensitivity reactions induced by exenatide in patients. One of them reported anaphylactic reaction in the patient with long acting release exenatide (2 mg once-weekly), namely, generalized urticarial, urticarial, uvular edema and vomiting.^[7]^ The second is a patient that suffered from generalized urticaria, and difficulty swallowing after the administration of exenatide.^[8]^ Although the third study reported an exenatide treatment with no allergic reactions because of well tolerated exenatide, the patient was allergic to exenatide by skin prick test and intradermal skin test, with the probable cause that drug skin test results was not always consistent with the clinical effects.^[9]^ The positive skin tests in all these studies and a basophil activation test with positive results in the first study forcefully supported an immunoglobulin E-mediated mechanism. Moreover, discontinuous treatment in our study may increase the risk of anaphylaxis. This is consistent with the second study that intermittent treatment of exenatide has developed an allergy,^[8]^ the possible cause is immunoglobulin E-mediated degranulation of mast cells.^[9]^ In addition, although off-label dose of exenatide in our study was mentioned, exenatide overdose has no clinical harm.^[10,11]^

Although anaphylaxis of exenatide has been reported, this is the first study that discusses the anaphylactic shock of exenatide reexposure in the patient who has interrupted exenatide treatment. Anaphylaxis, even anaphylactic shock of exenatide has not arose as frequent problems to date, which need us to paying more attention to its safety. Although we could not get permission to study the mechanism of anaphylactic shock further, we explore the possible allergic reactions, which can attract more attention of clinicians facilitating taking an effect treatment regimen.

## Author contributions

**Methodology:** Xujing Liu, Aihua Zhai.

Supervision: Bai Zhang.

Writing – original draft: Xujing Liu, Aihua Zhai.
